# Cytotoxicity mechanism of heterologous expression of phospholipase D in *Escherichia coli*

**DOI:** 10.1128/spectrum.03763-25

**Published:** 2026-06-22

**Authors:** Shaofeng Chen, Rongxin Wu, Xiaoyun Guo, Shuizhi Lin, Yinghua Lu, Xueping Ling

**Affiliations:** 1 Department of Chemical and Biochemical Engineering, College of Chemistry and Chemical Engineering, Xiamen University 12466, Xiamen, People's Republic of China; 2 State Key Laboratory of Microbial Metabolism, School of Life Sciences and Biotechnology, Shanghai Jiao Tong University 12474, Shanghai, People's Republic of China; 3 College of Chemical Engineering and Materials Science, Quanzhou Normal University 117823, Quanzhou, Fujian, People's Republic of China; 4 Xiamen Key Laboratory of Synthetic Biotechnology, Xiamen University 12466, Xiamen, People's Republic of China; 5 State Key Laboratory of Vaccines for Infectious Diseases, Xiang An Biomedicine Laboratory, Xiamen, People's Republic of China; Mikrobiologický ústav AV ČR, v. v. i., Třeboň, Czechia

**Keywords:** phospholipase D, cytotoxicity, phosphatidic acid, phospholipid, ion homeostasis

## Abstract

Phospholipase D (PLD) has multiple functions in cells and various industrial applications, including the production of high-purity and rare phospholipids, making it a highly valuable enzyme. However, the strong cytotoxicity of PLD and limited understanding of its mechanism in prokaryotic systems have resulted in low levels of heterologous PLD expression. In this study, we used a PLD-expressing strain model to characterize the morphological and physiological changes in PLD-exposed cells. By analyzing the intracellular ion content and cell membrane potential, we found that PLD-induced phosphatidic acid accumulation abnormally stimulated the continuous efflux of potassium ions through voltage-gated potassium channels. Disrupted ion homeostasis affects cell growth and metabolism, leading to a burst of reactive oxygen species levels and causing cellular damage. Transcriptomic analysis suggests that promoting the conversion of fatty acids to phospholipids, reducing phosphatidic acid accumulation, and minimizing phospholipid composition disruptions are key strategies for alleviating PLD cytotoxicity. This study highlights how PLD directly disrupts phospholipid homeostasis through its catalytic activity or indirectly affects cellular metabolism via phosphatidic acid as a signaling molecule. These findings are crucial for understanding the cellular mechanisms of PLD and enhancing heterologous expression levels.

**IMPORTANCE:**

This study provides valuable insights into the cellular mechanisms of enzyme cytotoxicity, using *Escherichia coli* expressing phospholipase D (PLD) as a model. By systematically analyzing physiological changes and transcriptomic profiles, it probes key factors such as ion homeostasis and membrane potential, offering a robust framework for understanding the barriers to heterologous protein expression. The discovery that PLD-induced accumulation of phosphatidic acid triggers continuous potassium efflux via voltage-gated channels underscores the critical link between disrupted ion balance and cell survival. These findings advance our understanding of PLD-mediated toxicity and pave the way for future metabolic engineering strategies designed to enhance the industrial production of this and other high-value enzymes.

## INTRODUCTION

Phospholipase D (EC 3.1.4.4, PLD), a member of the phospholipase superfamily, has been demonstrated to participate in cellular activities such as endocytosis, exocytosis, cell migration, mitosis (1), lipid droplet formation (2), and receptor internalization (3). It catalyzes two primary reactions: hydrolysis of phospholipids to produce phosphatidic acid (PA), and transphosphatidylation, which exchanges the polar head group of the substrate to form new phospholipids (4). Its unique transphosphatidylation activity enables the synthesis of high-value rare phospholipids, including phosphatidylserine (PS), 6-phosphatidyl-L-ascorbic acid, and phosphatidyl terpenes (5). While heterologous expression of PLD has advanced its industrial potential, severe cytotoxicity associated with its overexpression limits production yields, posing a significant challenge for industrial-scale manufacturing. Elucidating the specific mechanisms by which overexpressed PLD impacts microbial physiology is crucial for addressing this bottleneck (6).

Mishima et al. (7) observed that plasmids carrying the PLD gene were unstable in *Escherichia coli*, leading to rapid plasmid loss, particularly following induction. Tao et al. (8) hypothesized that rapid host cell death results from PLD-mediated degradation of membrane phospholipids, which destabilizes the cell membrane and causes lysis. Tao et al. (8) hypothesized that rapid host cell death results from PLD-mediated degradation of membrane phospholipids, which destabilizes the cell membrane and causes lysis. Conversely, Xiong et al. (9) demonstrated that a combinatorial strategy—integrating gene optimization, nutritional supplementation, and low-temperature induction—sustained high PLD expression in *E. coli* without inducing immediate cell death. This suggests that direct membrane lysis by PLD is not the primary cause of cell death.

PLD exerts both direct and indirect regulatory effects on the cell. It mediates the synthesis of key membrane components, such as phosphatidylethanolamine (PE) and PS, thereby influencing membrane morphology and properties. Additionally, PLD regulates cellular function through its catalytic product, PA, which serves as a pivotal signaling molecule (10, 11). We hypothesized that PLD interferes with the physiological activity of *E. coli* through distinct metabolic or signaling pathways. For instance, periplasmic overexpression of PLD in *E. coli* correlates with PA accumulation and altered metal ion concentrations (10). Similarly, during PLD secretion in *Bacillus brevis*, disruption of intracellular ion homeostasis leads to excessive Ca^2+^ accumulation and metabolic abnormalities (11). PLD may also play a role in microbial cells similar to that in animal and plant cells. Xiong et al. (10), along with our earlier studies (12), found that the overexpression of PLD in *E. coli* resulted in severe growth inhibition, while NaCl stress, or the mixed salt stress of NaCl and MgCl_2_, could alleviate the toxicity caused by PLD overexpression and promote an increase in PLD production. Several studies have shown that PLD uses phospholipids that make up the cell membrane as substrates and has a damaging effect on the cell membrane (10, 11, 13). These studies suggest that PLD overexpression disrupts ion homeostasis in cells, whereas salt stress helps restore a new ion balance, supporting PLD expression and providing insights into the cellular mechanisms of PLD overexpression. Investigating the upstream mechanisms of toxicity caused by overexpressed PLD in cells is conducive to the development of new optimized expression strategies.

In this study, we utilized an established PLD-overexpressing strain to investigate the mechanisms of PLD effects on *E. coli* cells. We conducted a comprehensive analysis involving cellular morphology, physiological status, ion content, membrane potential, PA levels, and transcriptomic profiling (RNA-seq). Our findings clarify the impact of PLD on phospholipid metabolism and provide metabolic engineering insights to overcome the physiological barriers hindering high-yield PLD production.

## MATERIALS AND METHODS

### Strains, plasmids, and chemicals

All the strains and plasmids used in this study are listed in Table S1. *E. coli* BW25113 *ΔtreA ΔtreC ΔtreF* (BW3) was used to express PLD (BW3-PLD). *E. coli* DH5α strain was used as the cloning host. Unless otherwise noted, chemicals were purchased from Sinopharm Chemical Reagent Co., Ltd. (Shanghai, China).

### Cultivation medium and conditions

Luria broth (LB) medium (10.0 g/L tryptone, 10.0 g/L NaCl, and 5.0 g/L yeast) was used to cultivate *E. coli* DH5α with 50 mg/L kanamycin (Kan) introduced in all cultures. BW3-PLD was incubated overnight in LB solid medium (LB medium with 1.5%–2% agar and 50 mg/L Kan) at 37°C, and the corresponding single colonies were incubated in LB medium with 50 mg/L Kan at 37°C and 200 rpm overnight. Then, 200 µL of the culture was transferred to 20 mL/250 mL TB medium (0.4% glycerol, 12 g/L tryptone, 24 g/L yeast, and 0.1 M potassium phosphate buffer) with 50 mg/L Kan and incubated at 37°C and 200 rpm for 5 h, and cell pellets were obtained by centrifugation (5,000*g*) at 4°C. The obtained cell pellets were resuspended in TB expression medium (0.6% glycerol, 12 g/L tryptone, 32 g/L yeast, 0.1 M potassium phosphate buffer, and mixed salt of 0.1 M MgCl_2_ and 0.4 M NaCl, pH 6.9) and cultured at 18°C and 200 rpm for 8 h; 0.15% (wt/vol) arabinose was added at the start and after 2 h.

To determine whether the beneficial effect of mixed salt was a specific countermeasure to PLD toxicity or a generic osmotic adaptation, we compared the physiological responses across four experimental conditions. The experiments were divided into four groups. PLD + Salt: strain BW3 was cultured in TB expression medium with the addition of inducer and mixed salt; PLD: strain BW3 was cultured in TB expression medium with the addition of inducer and without salt; Control + Salt: strain BW3 was cultured in TB expression medium with mixed salt and without addition of inducer; Control: strain BW3 was cultured in TB expression medium without addition of inducer and salt. Unless otherwise noted, samples were incubated for 0, 4, and 8 h after induction.

### Cell morphology observation

Scanning electron microscopy (SEM, Sigma, Zeiss) was used to examine cell morphology. The cells cultured for 8 h were collected by centrifugation and washed with 1× PBS solution. The cells were then resuspended in 2.5% glutaraldehyde solution for low-temperature fixation, followed by centrifugation and resuspension in 1× PBS. The suspensions were fixed on silicon wafers with a fulvalene membrane using a 2.5% glutaraldehyde solution. After washing and drying, the silicon wafers were coated with platinum using a sputter coater and observed under SEM to assess cell morphology.

### Cell membrane phospholipid and protein analysis

The Bligh-Dyer method was used to extract phospholipids from cell membranes, and thin-layer chromatography was used to analyze phospholipids. The thin-layer chromatography results for the standard samples are shown in Fig. S1. Membrane protein extraction was performed using a membrane protein extraction kit (SolarBio), and the membrane protein components were analyzed by SDS-PAGE.

### Determination of ATP content

The ATP content was determined using the CellTiter-Glo Cell Viability Assay Kit (Promega). The samples were processed according to the manufacturer’s instructions, and the results were determined using a microplate reader (SpectraMax MS, Molecular Devices). The results are expressed as fluorescence intensity (Relative Light Units [RLUs]), where a higher fluorescence intensity indicates a higher ATP content.

### Analysis of cell membrane permeability

Referring to the method of cell membrane permeability determination mentioned in the previous literature (10) with slight adjustments, the organisms were stained using SYTOX chlorocyanine dead cell nucleic acid dye (Keygen Biotech). Cell membrane permeability was qualitatively analyzed based on the intensity of the emitted fluorescence. The measurements were normalized to relative membrane permeability, with larger values representing greater cell permeability.

### Determination of malondialdehyde content

The method for the determination of malondialdehyde (MDA) in *E. coli* was adapted from Wang et al. (14). The samples were washed with 1× PBS, and equal proportions of 0.6% 2-thiobarbituric acid were added and boiled for 0.5 h. After centrifugation, the supernatants were collected for the determination of *A*_600_, *A*_532_, and *A*_450_. The MDA concentration was calculated using the formula described in the literature. The measurements were normalized to relative MDA content, with larger values representing a higher MDA content.

### Measurement of reactive oxygen species content

Reactive oxygen species (ROS) content was determined using a reactive oxygen detection kit (Solarbio). The samples were processed according to the manufacturer’s instructions, and the results were determined using a microplate reader (SpectraMax MS, Molecular Devices). The results were expressed as 2′,7′-dichlorodihydrofluorescein diacetate (DCFH-DA) fluorescence (A.U.), and a higher fluorescence intensity indicated a higher ROS content.

### Measurement of intracellular ion content

The cells were washed with 1× PBS. After drying and weight determination, nitric acid was added to dissolve the samples. The solvent was evaporated, and 2% dilute nitric acid was added to dissolve and determine the ion content using an Inductively Coupled Plasma Emission Spectrometer (Thermo Fisher Scientific).

### Analysis of cell membrane potential

The method for the determination of the membrane potential of *E. coli* using Thioflavin T (ThT) was adapted from a previous report (15). The samples were diluted with LB medium until the OD_600_ reached approximately 0.1. ThT was added to a final concentration of 10 μM and incubated at room temperature for 10 min. Samples were set up in multiple sets of parallel, and the fluorescence intensity of the samples and blank samples was measured using a microplate reader (SpectraMax MS, Molecular Devices).

### Phosphatidic acid extraction and determination

PA extraction and content determination were performed using a Total Phosphatidic Acid Assay Kit (Cell Biolabs). Samples were collected according to the manufacturer’s instructions, and the results were determined using a microplate reader (SpectraMax MS, Molecular Devices).

### Enzyme extraction and assay

The PLD extraction and activity assays were performed as described previously (9). Cells were harvested by centrifugation, and the supernatant was collected as the extracellular PLD. For intracellular PLD, the cell pellets were resuspended in an extraction buffer containing 200 mM Tris-HCl (pH 7.5), 10 mM EDTA, and 0.05% (wt/vol) sodium deoxycholate. The suspension was incubated at room temperature for 1 h with intermittent vortexing, followed by centrifugation to collect the supernatant.

PLD activity was determined by measuring choline release. The substrate solution consisted of 10 mM phosphatidylcholine (PC) emulsified in 5% (wt/vol) Triton X-100. The reaction mixture, containing the substrate, 0.1 M Tris-maleate-NaOH (pH 5.5), 2.5% Triton X-100, and 10 mM CaCl_2_, was incubated with the enzyme at 37°C for 10 min. The reaction was terminated by adding a stop solution (1 M Tris-HCl [pH 8.0] and 0.5 M EDTA) and boiling. Released choline was quantified by adding a detection solution containing choline oxidase (1.25 U), peroxidase (1 U), 4-aminoantipyrine (1 µmol), and phenol (1.6 µmol) in 0.1 M Tris-HCl buffer (pH 8.0). After incubation at 37°C for 1 h, the mixture was diluted with 1% Triton X-100, and absorbance was measured at 500 nm. One unit (U) of activity was defined as the amount of enzyme releasing 1 μmol of choline per minute.

### RNA-seq and transcriptomics analysis

Samples were taken from the broth after 4 h of cultivation in TB expression medium and sent to Sangon Biotech for transcriptome data analysis. Annotation classification and enrichment analysis were performed based on information obtained from the Kyoto Encyclopedia of Genes and Genomes (KEGG), Gene Ontology, and Cluster of Orthologous Groups public protein databases.

### Data analysis

Values are shown as mean ± standard deviation from three biological replicates. The results are marked with different letters if *P* < 0.05.

## RESULTS

### Effect of PLD overexpression on cell morphology, cell membrane phospholipids, and membrane proteins

**Fig 1:**
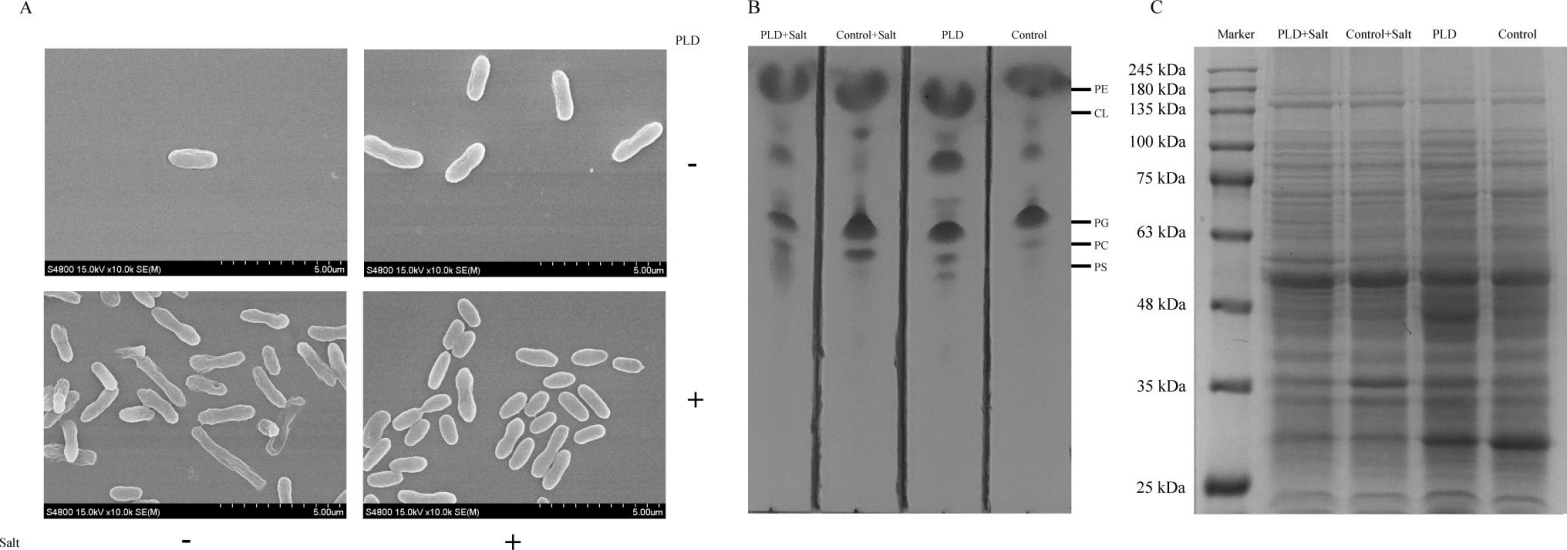
Cell membrane characterization of the strains. SEM characterization of cell morphology (**A**), cell membrane phospholipids (**B**), and cell membrane proteins (**C**). Salt +, subjected to mixed salt stress; Salt −, no mixed salt stress; PLD +, PLD expression (addition of inducer arabinose); PLD −, no PLD expression (without addition of inducer arabinose); CL, cardiolipin; PC, phosphatidylcholine; PS, phosphatidylserine; PG, phosphatidylglycerol; and PE, phosphatidylethanolamine.

In our previous study, we found that PLD overexpression in *E. coli* resulted in severe growth inhibition, and the application of mixed salt stress alleviated the toxicity of PLD and promoted an increase in PLD production (12). As shown in Fig. 1A, the cells of the Control and Control + Salt groups exhibited similar morphology, with a smooth, rod-like shape. In contrast, cells overexpressing PLD without mixed salt stress (PLD group) displayed significant abnormalities, including cell wrinkling, irregular length, and cell rupture. However, cells overexpressing PLD under mixed salt stress (PLD + Salt group) regained their normal morphology, suggesting that mixed salt stress could mitigate the toxic effects of PLD on cells.

The major phospholipids in the *E. coli* membrane are PE, phosphatidylglycerol (PG), and cardiolipin (CL) (16). Compared with the Control group, the PLD group showed an increase in CL, PC, and PS (Fig. 1B), whereas the PLD + Salt group exhibited a decrease in CL and PG. This differed from the PG increase and CL decrease observed in the Control + Salt group compared with the control group, implying that PLD expression and mixed salt stress primarily interact by regulating anionic phospholipid CL and PG synthesis. PG and CL are crucial for maintaining normal cell morphology (17). Disruptions in phospholipid composition can affect cell membrane fluidity and rigidity. Therefore, PLD-overexpressing cells exhibited morphological abnormalities (Fig. 1A). Mixed salt stress counteracts phospholipid disturbances caused by PLD catalytic activity, normalizing cellular phospholipid composition and reducing metabolic impacts.

Membrane proteins are the key components of *E. coli* cell membranes. Approximately 500,000 membrane protein molecules reside in the cytoplasmic membrane and participate in diverse enzymatic reactions (18). PLD expression altered the membrane protein profile of *E. coli*, noticeably affecting bands around 48 kDa. However, under mixed salt stress, the membrane protein composition showed a different pattern compared to the PLD group. Previous studies have indicated that the absence of phospholipid components can lead to improper folding and expression of outer membrane proteins (17), and salt stress is known to suppress the expression of certain *E. coli* outer membrane proteins with smaller pores (19). Our results suggest that the combined effect of PLD expression and salt stress leads to remodeling of the membrane protein composition, likely as an adaptive response to phospholipid perturbations and osmotic pressure, rather than a simple restoration of the control profile.

### Effect of PLD overexpression on cell status

**Fig 2:**
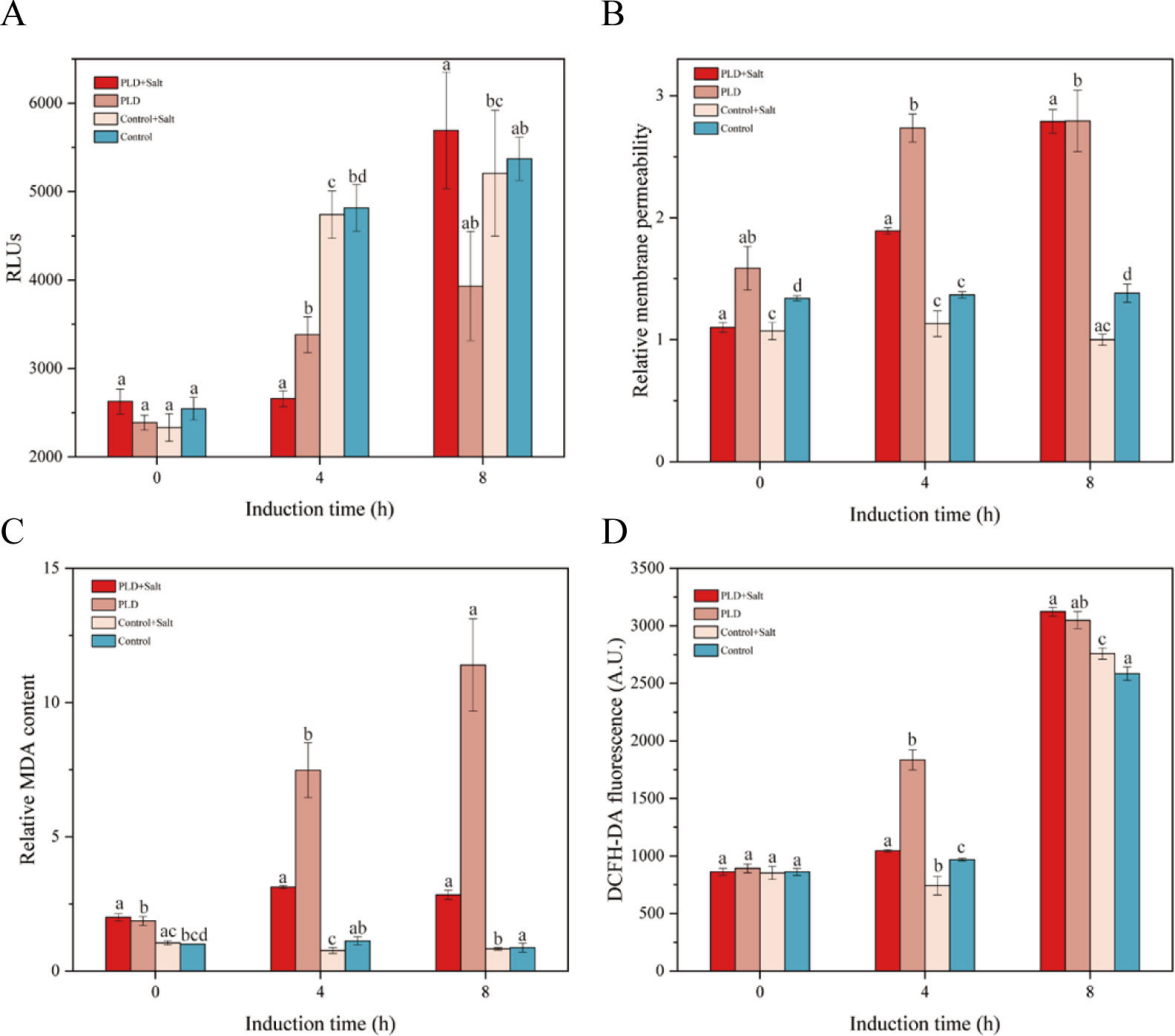
ATP content (**A**) measured in RLUs; relative membrane permeability (**B**); MDA content (**C**), an indicator of lipid peroxidation; ROS accumulation (**D**) indicated by DCFH-DA fluorescence intensity (A.U., Arbitrary Units).

The cellular damage caused by PLD overexpression can be characterized by cellular parameters, including ATP levels, membrane permeability, membrane lipid oxidation markers, and ROS, which can help to further understand the role of PLD overexpression in cells. ATP is essential for DNA replication, biosynthesis, protein assembly, and substance transport and serves as bioenergy for many cellular reactions. The maintenance of cellular homeostasis requires a constant supply of ATP (20). Therefore, ATP levels reflect the state of the cell and serve as an indicator of cellular vitality. Figure 2A shows that the cells were in a state of relatively low vitality immediately. As fermentation proceeded, the increasing ATP concentration indicated growing cellular vitality. The two groups of cells without PLD expression presented similar ATP levels at 4 and 8 h, indicating that mixed salt stress did not affect ATP levels in cells. However, overexpression of PLD led to a reduced ATP concentration at 4 h, which may be due to the rapid expression of PLD in the first 4 h, consuming more ATP. At 8 h, the ATP levels in the PLD group showed a small increase, while those in the PLD + Salt group showed a rapid improvement and surpassed those of the other three groups. This means that PLD overexpression leads to a decrease in cellular ATP levels and affects normal cellular metabolic activities, which could be effectively alleviated by salt stress, resulting in the rapid recovery of cellular vitality.

Membrane permeability is a key indicator of cell membrane functionality. Xiong et al. (10) reported that overexpression of PLD resulted in a progressive increase in membrane permeability over time. Figure 2B shows that both the Control and the Control + Salt groups maintained constant membrane permeability, indicating that the cell membrane functions normally to ensure cellular homeostasis. In contrast, overexpression of PLD without salt stress led to an obvious increase in membrane permeability within the first 4 h, whereas salt stress effectively mitigated the increase in membrane permeability. After 8 h of expression, the value in the PLD + Salt group was as high as that in the PLD group. These results indicate that rapid PLD expression induces severe damage to cell membranes. Although salt stress could delay the process of PLD damage to cells, it did not alter the final extent of PLD for cell membrane disruption. Finding a method that can effectively inhibit PLD cytotoxicity may be the best strategy for improving cell viability.

During cell aging and cell death, structural components and organelles often undergo oxidation. Malondialdehyde is a by-product of membrane lipid oxidation, and its levels reflect the extent of lipid oxidation in the cell membrane (21). As shown in Fig. 2C, MDA content in the two control groups (with or without salt stress) was consistently low. In contrast, the PLD group exhibited much higher MDA levels than normal cells, and these levels increased over time. When mixed salt stress was applied to PLD-expressing strains, the MDA content was significantly reduced and maintained at a relatively stable level during the process. These results suggested that PLD expression worsens membrane oxidation, which can be effectively inhibited by mixed salt stress.

Membrane peroxidation is often associated with abnormal ROS levels (22). As plotted in Fig. 2D, ROS in normal cells remained at low levels until the end of fermentation, but there was no oxidative damage in the late-stage cells (low MDA content in Fig. 2C), indicating that cells can also maintain normal metabolic activities (Fig. 2B). PLD overexpression resulted in an obvious increase in ROS levels during the first 4 h, which was effectively reduced under salt stress, helping the cells maintain ROS homeostasis and protecting them from oxidative damage (low MDA content in Fig. 2C). At 8 h, all the groups generated large amounts of ROS. This indicates that the strains had lost their optimal protein expression state, and the high level of ROS reflects the abnormal cell state.

### Effect of PLD expression on the intracellular ion content and cell membrane potential

**Fig 3:**
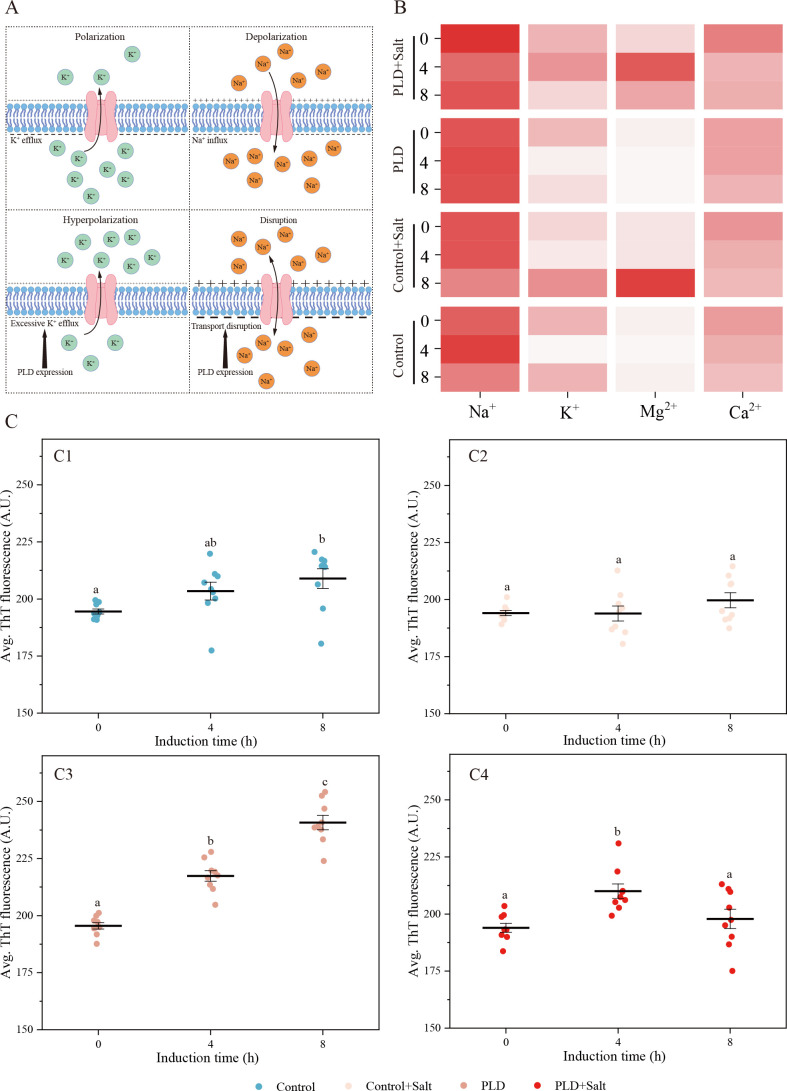
Schematic illustration of Na^+^ and K^+^ fluxes during polarization, depolarization, and hyperpolarization, showing the proposed mechanism of PLD-induced transport disruption based on the data in this study and reference 24 (**A**); intracellular ion content heatmap (**B**), red and white represent higher and lower values, respectively; membrane potential, the higher fluorescence intensity means the greater the degree of polarization (**C**).

Cells utilize the Na^+^ and K^+^ gradients across the cell membrane for various physiological processes, as the ion gradient across the membrane stores a significant amount of energy (23). Maintaining a normal membrane potential requires cells to transport and adjust to an appropriate Na^+^ and K^+^ gradient, which is crucial for many enzymatic functions (Fig. 3A). Additionally, Mg^2+^ and Ca^2+^ are crucial for cell activity and can interact with Na^+^ and K^+^. As shown in Fig. 3B, for the control group cells, Na^+^ levels first increased and then decreased, while K^+^ levels exhibited the opposite trend, first decreasing and then increasing. Mg^2+^ levels remained relatively low and stable, and Ca^2+^ was continuously expelled from the cell. When mixed salt stress was applied to the control group, it remarkably increased Na^+^ levels at 0 h and caused large amounts of K^+^ and Mg^2+^ to enter the cell at 8 h, but had no obvious effect on Ca^2+^ content. In the PLD group, compared to the control group, Na^+^ levels remained relatively high, with no significant fluctuation during the process. K^+^ levels obviously decreased at 8 h, Mg^2+^ levels were slightly lower during the process, and Ca^2+^ expulsion was hindered to some extent at 4 h. When mixed salt stress was applied in the PLD group, Na^+^ and Ca^2+^ showed an obvious increase at 0 h and a decrease at 4 h, K^+^ showed a clear enhancement at 4 h, while a large accumulation of Mg^2+^ was observed during the whole process, especially at 4 h. It can be seen that the ion perturbation caused by the PLD overexpression mainly involves Na^+^ and K^+^, which can be regulated by Mg^2+^.

An imbalance in intracellular ion homeostasis can disrupt enzymatic reactions and alter cell membrane potential. The accumulation of the positively charged fluorescent dye ThT is dependent on the membrane potential (15). Therefore, an increase in fluorescence intensity indicates a hyperpolarization event. As shown in Fig. 3C, normal cells showed slight polarization over time (Fig. 3C1), whereas mixed salt stress reduced the degree of membrane polarization (Fig. 3C2). Compared to the control group, PLD overexpression led to membrane-sustained hyperpolarization (Fig. 3C3), while mixed salt stress effectively suppressed the hyperpolarization trend in PLD-overexpressing strains (Fig. 3C4). The generation of membrane action potentials requires K^+^ (25) for depolarization, so cells will take up high levels of K^+^ to maintain normal biological functions. Thus, persistently low K^+^ levels in the PLD group (Fig. 3B) may explain the observed membrane hyperpolarization.

### Analysis of PA content and voltage-gated K^+^ channel

**Fig 4:**
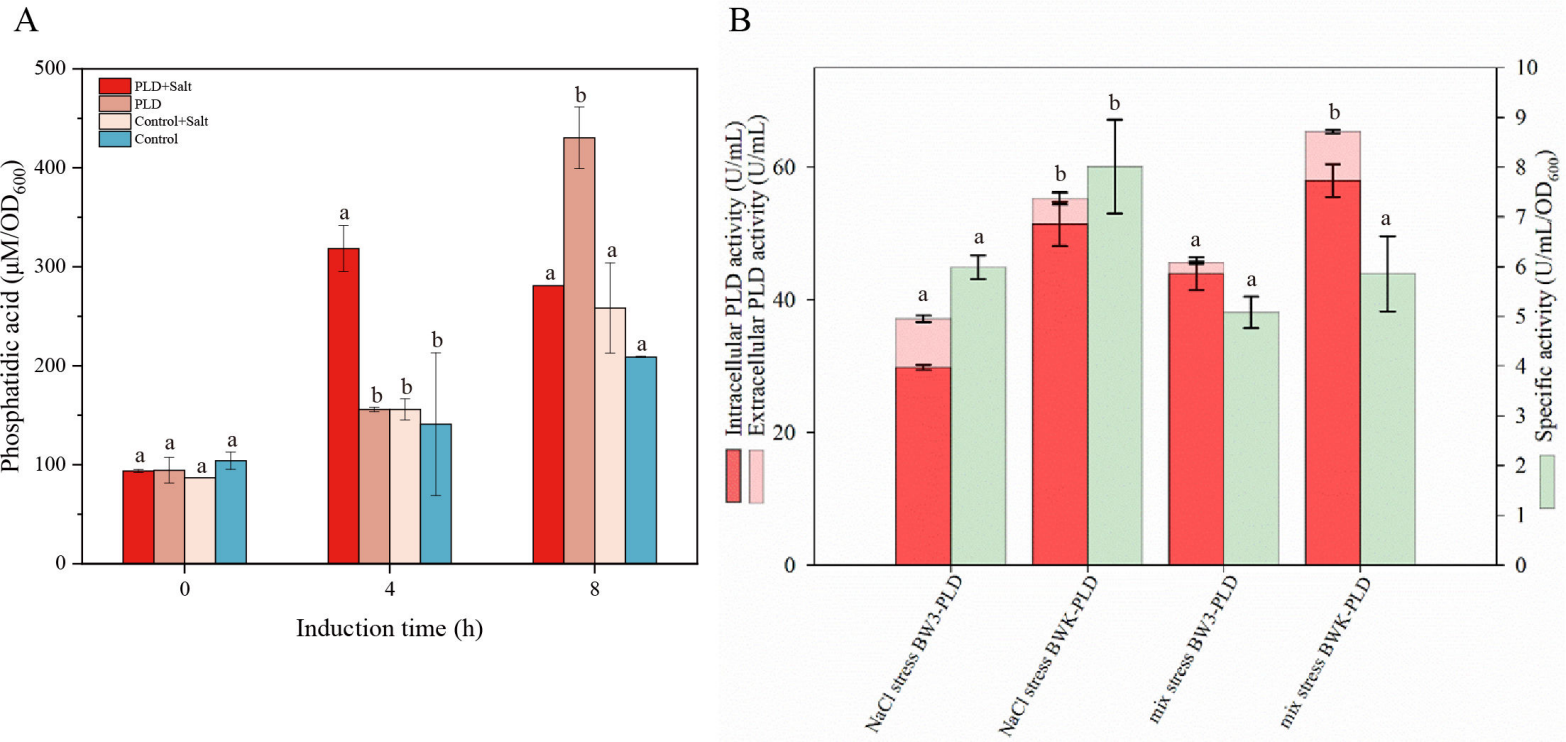
PA content of strains (**A**); the measurements were divided by the corresponding OD_600_ to represent the PA content per unit cell; PLD expression results of BW3-PLD and BWK-PLD (**B**); NaCl stress, 0.5 M NaCl; mix stress, 0.1 M MgCl_2_ and 0.4 M NaCl. BWK was constructed on the basis of BW3 by the CRISPR gene editing method in the previous study, which was accomplished using pCas and pTargetF-*kch*.

Maintaining high intracellular K^+^ levels is essential in bacteria, and ion homeostasis depends on specific ion channels and transport proteins (26). In a study by Stratford et al. (27), electrical stimulation of *E. coli* cells led to K^+^ efflux and membrane hyperpolarization, indicating the presence of voltage-gated K^+^ channels in *E. coli*. It has been reported that PLD2-catalyzed production of PA enhances the activity of the voltage-gated K^+^ channels TREK1 and TREK2 (28). Therefore, PA may be one of the critical signaling molecules in voltage-gated K^+^ channels. As shown in Fig. 4A, in the early stages of induction (4 h), all groups except the PLD + Salt group had higher PA levels, while the other three groups had similar PA levels, which may be induced by rapidly elevated PLD expression (Fig. S2). However, in the later stages (8 h), the cells of the PLD group accumulated significantly higher levels of PA, and the PLD + Salt group presented reduced PA content after metabolic adjustments. PLD overexpression without salt stress led to a final increase in PA content, which may excessively stimulate K^+^ channels to disrupt ionic homeostasis and lead to abnormal membrane potential.

The Kch K^+^ channel has a typical voltage-gated potassium channel topology consisting of six transmembrane segments and a pore region located between the fifth and sixth helices, which shows significant similarity to the corresponding region in eukaryotic potassium channels (29). We hypothesize that the excess PA generated in cells exposed to PLD may stimulate or be associated with the activation of Kch, a putative voltage-gated K^+^ channel, leading to abnormal intracellular potassium levels. Therefore, Kch was knocked out in the BW3-PLD strain to assess its effect on PLD overexpression. As shown in Fig. 4B, the absence of Kch resulted in 48.78% and 43.33% increases in PLD production under mixed salt and NaCl stress, respectively. The significantly elevated expression of PLD suggests that Kch is a key target for PLD-induced cellular toxicity.

### Comparative transcriptomics of PLD expression strains

The strains expressing PLD with and without salt stress showed different physiological states, and transcriptomic analysis can be performed to understand the gene expression patterns and self-rescue modes of cells under PLD stress. Given that salt stress had a relatively minor effect on normal cells, PLD reached its maximum yield after 8 h of induction. We used three groups of cells: control (C), PLD (P), and PLD + Salt (SP) at 4 h of induced culture to assess transcriptomic differences (P vs C and SP vs P).

**Fig 5:**
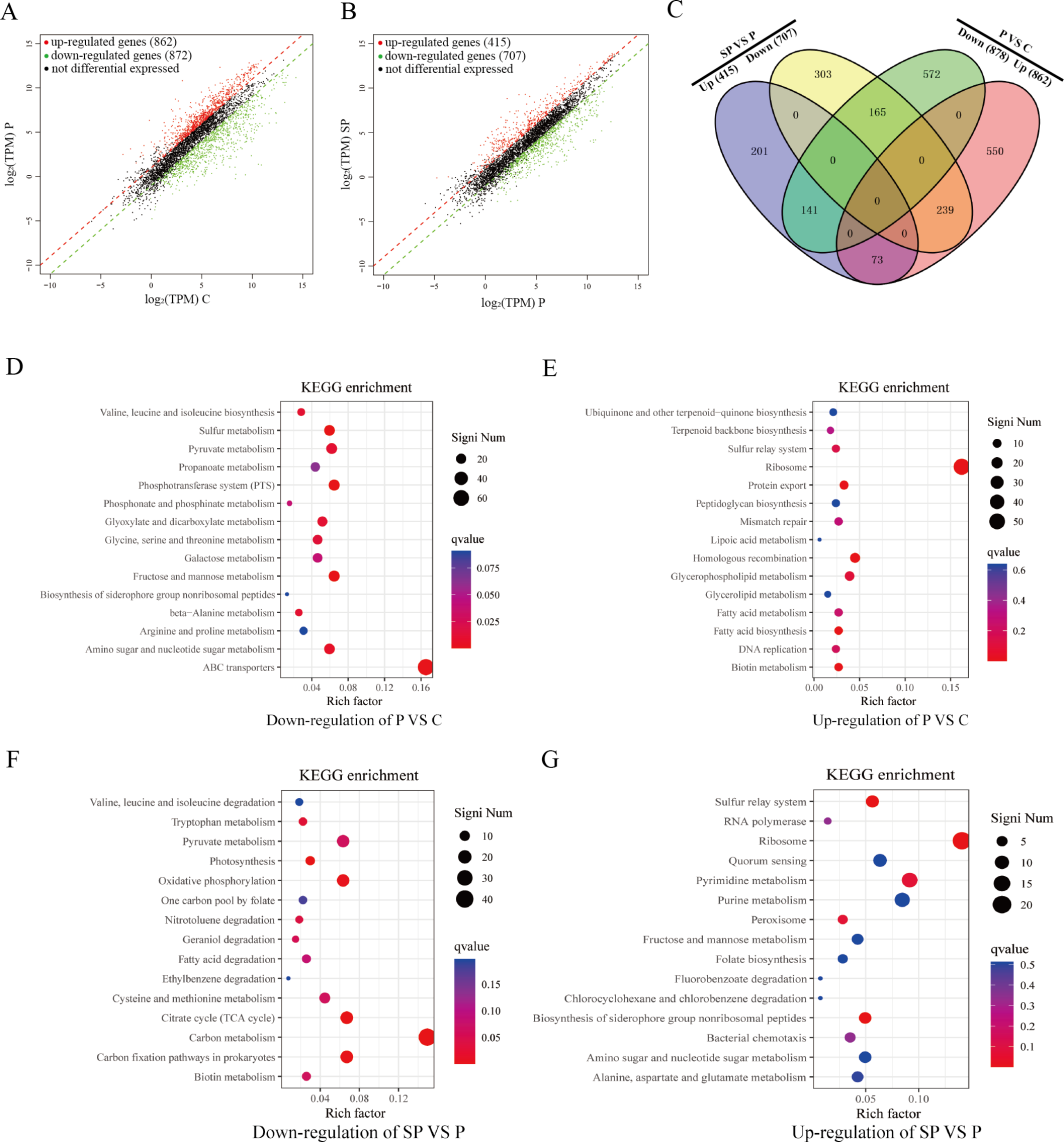
Results of transcriptome comparison scatter plots of sample expression difference, P vs C (**A**) and SP vs P (**B**); numbers of upregulated and downregulated genes in different groups (**C**); KEGG annotation enrichment analysis of downregulation of P vs C (**D**), upregulation of P vs C (**E**), downregulation of SP vs P (**F**), and upregulation of SP vs P (**G**).

Statistical analysis of the transcriptomic data in Fig. 5A (P vs C) and Fig. 5B (SP vs P) revealed significant differential gene expression between the two groups. Comparative analysis (Fig. 5C) revealed that the expression of 1,740 genes in the P group differed significantly from that in the C group, with 862 genes upregulated and 878 genes downregulated. Likewise, the SP group showed different expressions of 1,122 genes compared to the P group, with 415 genes upregulated and 707 genes downregulated. By comparing the genes that were significantly upregulated and downregulated in both groups, it was found that 73 genes were upregulated, and 165 genes were downregulated, highlighting the significant impact of PLD overexpression on cellular metabolism (Fig. 5C).

To further investigate the effect of PLD overexpression on cellular gene expression, KEGG pathway enrichment analysis was performed (*P* < 0.05). Compared with the C group, the downregulated pathways in the P group (Fig. 5D) included sulfur metabolism, fructose and mannose metabolism, phosphotransferase system, ABC transporters, amino sugar and nucleotide sugar metabolism, valine/leucine/isoleucine biosynthesis, glyoxylate and dicarboxylate metabolism, β-alanine metabolism, pyruvate metabolism, glycine/serine/threonine metabolism, and galactose metabolism. The upregulated pathways in Group P (Fig. 5E) included ribosome, fatty acid biosynthesis, protein export, biotin metabolism, glycerophospholipid metabolism, homologous recombination, sulfur relay system, DNA replication, and mismatch repair. Compared with the P group, the downregulated pathways in the SP group (Fig. 5F) include citrate cycle, carbon metabolism, carbon fixation pathways in prokaryotes, tryptophan metabolism, nitrotoluene degradation, cysteine and methionine metabolism, pyruvate metabolism, fatty acid degradation, valine/leucine/isoleucine degradation, lysine degradation, benzoate degradation, and glycerophospholipid metabolism; the upregulated pathways in Group SP (Fig. 5G) include ribosome, biosynthesis of siderophore group nonribosomal peptides, sulfur relay system, peroxisome, pyrimidine metabolism, bacterial chemotaxis, and RNA polymerase. Pyruvate metabolism was downregulated in both PLD-expressing groups (P group and SP group) compared with the C group; similarly, the upregulated pathways included the ribosome and sulfur relay system. This indicates that overexpression of PLD promotes ribosome-related metabolic pathways and interferes with pyruvate and its downstream metabolism, as well as activates genes related to detoxification and antioxidant defense. Moreover, the P group exhibited increased expression of fatty acid synthesis genes compared to the C group, whereas the SP group showed decreased expression of fatty acid degradation genes relative to the P group. This suggests that changes in fatty acid metabolism may be metabolic rescue measures in response to PLD stress, which is closely linked to phospholipid metabolism.

### Analysis of glycerophospholipid metabolic pathways

**Fig 6:**
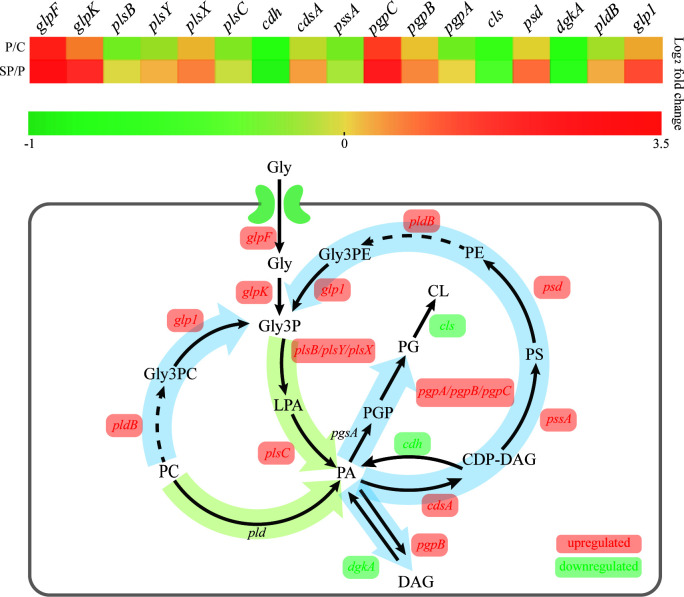
Comparison of transcription levels of glycerophospholipid metabolic pathways. Gly, glycerol; Gly3P, glycerol-3-phosphate; LPA, lysophospholipid; PA, phosphatidic acid; Gly3PC, glycerol-3-phosphocholine; PC, phosphatidylcholine; Gly3PE, glycerol-3-phosphoethanolamine; PE, phosphoethanolamine; PS, phosphatidylserine; CDP-DAG, CDP-diacylglycerol; DAG, diacylglycerol; PGP, phosphatidylglycerol; PG, phosphatidylglycerol; CL, cardiolipin. glpF, glycerol uptake facilitator protein; glpK, glycerol kinase; plsB, glycerol-3-phosphate 1-O-acyltransferase; plsY, glycerol-3-phosphate 1-O-acyltransferase; plsX, phosphate acyltransferase; plsC, lysophospholipid transporter; cdh, CDP-diacylglycerol diphosphatase; cdsA, phosphatidate cytidylyltransferase; pssA, CDP-diacylglycerol-serine O-phosphatidyltransferase; pgpC, phosphatidylglycerophosphatase C; pgpB, phosphatidylglycerophosphatase B; pgpA, phosphatidylglycerophosphatase A; cls, cardiolipin synthase; psd, phosphatidylserine decarboxylase; dgkA, diacylglycerol kinase; pldB, lysophospholipase L2; and glp1, glycerophosphodiester phosphodiesterase.

The glycerophospholipid metabolic pathways are significantly affected by PLD overexpression (30), and exploring changes in this pathway may help identify strategies for regulating phospholipid homeostasis under PLD overexpression. As shown in Fig. 6, PLD overexpression disrupted the glycerophospholipid pathway, primarily affecting PA, and most genes in this pathway were upregulated. This phenomenon may result from PLD catalyzing the conversion of PC and other phospholipids into excess PA, which must be rapidly converted into other phospholipids to minimize its effects on the cell. Additionally, excess phospholipids tend to be converted into neutral lipid DAG or recycled into glycerol-3-phosphate. Anionic phospholipids play critical roles in cellular activity (31, 32), and gene transcription levels respond differently to anionic phospholipids, promoting PG synthesis, while inhibiting CL production. PA was the precursor for the synthesis of PG, which in turn was the precursor for the synthesis of CL. The differential transcription of these two anionic phospholipids appears to stem from PLD-induced disturbances in phospholipid composition, suggesting that PLD may have different effects on the two phospholipids. Under mixed salt stress, transcriptional changes associated with PLD expression mirror those observed under PLD expression without salt stress, suggesting that this metabolic pattern is an effective strategy for mitigating PLD-induced cytotoxicity.

## DISCUSSION

PLD has wide applications in the production of high-value phospholipids. However, many strategies have been ineffective in remarkably enhancing the heterologous expression levels of PLD owing to its cytotoxicity. Therefore, we employed various methods to characterize the endogenous mechanisms of PLD inducing cytotoxicity in *E. coli*. Overexpression of PLD fundamentally alters membrane phospholipid composition. Anionic phospholipids, specifically PG and CL, are critical for the translocation, stability, and function of membrane protein complexes (31, 33). Our results indicate that the PLD-mediated depletion of these lipids leads to a global remodeling of the membrane proteome, likely due to impaired protein assembly and folding. Consequently, cells exposed to PLD exhibited morphological abnormalities, increased permeability, and lysis. These phenotypic changes suggest that PLD toxicity is not merely physical membrane disruption but a systemic metabolic failure triggered by the loss of membrane integrity and subsequent oxidative damage to cellular components.

High ATP levels typically stimulate ROS production (34), as observed in the control group (Fig. 2A and D). However, PLD overexpression disrupted this homeostasis, causing premature lipid peroxidation (indicated by elevated MDA) and damage to intracellular macromolecules (35). This suggests that PLD-induced membrane perturbation impairs the electron transport chain—likely through the displacement of CL required for respiratory complexes (17)—leading to uncontrolled ROS generation and metabolic collapse. Mixed salt stress significantly mitigated this oxidative damage (Fig. 2C and D). Unlike a generic osmotic response, we propose that the high ionic strength specifically counteracts the PLD-induced defects, stabilizing membrane protein complexes and helping restore the ionic balance required for efficient ROS detoxification.

Regulating Na^+^/K^+^ balance is fundamental for ATP synthesis and enzyme activation in *E. coli* (36, 37). PLD overexpression severely disrupted this balance, resulting in abnormally high intracellular Na^+^ and consistently depleted K^+^ levels. While mixed salt stress allows cells to establish a viable ionic equilibrium, the persistent K^+^ loss in PLD-expressing cells points to a specific transport defect. Critically, this depletion appears to be driven by active transport mechanisms rather than passive leakage through a damaged membrane. Comparisons of ion flux under salt stress suggest that the external cations may compete with or stabilize the transport machinery, thereby preventing the pathological efflux of K^+^ induced by PLD.

Based on the intracellular ion content changes (Fig. 3B), the persistently low K^+^ levels in the PLD group could explain the observed membrane hyperpolarization. Membrane potential in *E. coli* is dynamic and regulates a wide range of physiological processes, such as pH homeostasis, membrane transport, motility, and cell division (38). Membrane hyperpolarization can impair the ability to transmit ions or compromise the membrane integrity. Additionally, intracellular ATP production depends on depolarization events involving K^+^ channels (39). Abnormal membrane potential can hinder the proliferation and growth of *E. coli*. Thus, PLD overexpression induces membrane hyperpolarization, which disrupts cellular homeostasis (ROS and ion balance), damages the membrane, and reduces ATP synthesis, ultimately impairing cell growth and proliferation. The cytotoxicity of PLD may primarily stem from membrane hyperpolarization, triggering a cascade of energy and metabolic disruptions in the cell. A comparison of the cell membrane potentials of the two groups (in 4 h) under mixed salt stress (Fig. 3C2 and C4), along with their corresponding PA levels, revealed a correlation between PA accumulation and cell membrane polarization. A comparison of the 8 h non-mixed salt stress groups (Fig. 3C1 and C3) led to the same conclusion. While it is established in eukaryotes that PA can stimulate voltage-gated potassium channels, we infer that an analogous effect exists in *E. coli*. The expression of PLD was significantly upregulated in the *kch* knockout strain, suggesting Kch is involved in maintaining intracellular K^+^ levels in PLD-exposed cells. These results suggest a potential link between PLD activity, PA accumulation, and Kch-mediated ion homeostasis. It should be noted that while Kch possesses structural features characteristic of voltage-gated channels (29), direct electrophysiological evidence for its gating mechanism in *E. coli* is still lacking due to the technical challenges of bacterial patch-clamping and the absence of Kch-specific inhibitors. Therefore, the proposed activation of Kch by PA is an inference based on our genetic data and the observed correlation between PLD activity and potassium shifts. Future studies utilizing reconstituted systems may provide more direct biophysical evidence for this lipid-protein interaction.

Changes in transcript levels in PLD-exposed cells were demonstrated using RNA-seq. Downregulated genes were primarily involved in transport, carbon, and energy metabolism, suggesting that PLD overexpression inhibits cellular carbon utilization and reduces ATP supply. Conversely, upregulated genes were linked to DNA replication, protein folding, and lipid metabolism, reflecting a stress response likely connected to uncontrolled ROS production. Under mixed salt stress, genes related to intracellular substance transport and ROS clearance were significantly upregulated. We propose that the rescue effect of mixed salts is twofold: transcriptionally, it upregulates stress-response genes; biophysically, the high concentration of cations likely stimulates intracellular ion redistribution and stabilizes cell membranes. Additionally, changes in the phospholipid metabolism pathway induced by mixed salt stress indicated that restricting the glycerophospholipid metabolic pathway can effectively maintain phospholipid homeostasis. Therefore, reprogramming the glycerophospholipid metabolic network to redirect abnormal phospholipid flux toward DAG is expected to create a favorable environment for PLD expression. Overall, the perturbation of membrane phospholipids, along with changes in transcription levels, suggests that regulating PA-related pathways and membrane repair provides targets for enhancing PLD tolerance.

### Conclusion

This study reports on the cellular state and mechanism of PLD-exposed prokaryotic cells. For the first time, we discovered that PLD overexpression in *E. coli* led to decreased K^+^ levels and cell membrane hyperpolarization, thereby hypothesizing that increased PA content continuously stimulates voltage-gated potassium channels to export K^+^, resulting in cellular metabolic disorders, meaning that PA, Kch of the potential voltage-gated K^+^ channel, and glycerophospholipid metabolism pathway may be key targets of PLD toxicity. This study provides new insights into the mechanism of PLD cytotoxicity and offers a theoretical basis for alleviating the toxicity. Based on these findings, we propose several specific strategies to achieve high-level heterologous expression of PLD: (i) metabolic engineering to accelerate the conversion of accumulated PA into downstream phospholipids to restore lipid homeostasis; (ii) targeted deletion or modification of the *kch* gene to block the pathway of potassium loss; and (iii) optimization of fermentation conditions, such as ion supplementation, to stabilize the membrane potential.

## Data Availability

Data will be made available on request.

## SUPPLEMENTAL MATERIAL

Supplemental material spectrum.03763-25-s0001.docx Table S1; Fig. S1 and S2.

ASM does not own the copyrights to Supplemental Material that may be linked to, or accessed through, an article. The authors have granted ASM a non-exclusive, world-wide license to publish the Supplemental Material files. Please contact the corresponding author directly for reuse.
