# Supplementary material for: Cytotoxicity mechanism of heterologous expression of phospholipase D in Escherichia coli
Source: Microbiol Spectr. 2026 Jun 22;14(8):e03763-25. doi: 10.1128/spectrum.03763-25 (PMC13436071; doi:10.1128/spectrum.03763-25)
Supplement: Supplementary file 1 [file spectrum.03763-25-s0001.docx]

Table S1 Strains and plasmids

| Strains and plasmids | Parent/genotype | Source |
| --- | --- | --- |
| Strains |  |  |
| *E. coli* BW25113 (BW) | *Δ(araD-araB) 567 ΔlacZ4787(::rrnB-3) λ^-^*  *rph-1 Δ(rhaD-rhaB)568 hsdR514* | Lu lab |
| BW3 | [BW25113] *ΔtreA ΔtreC ΔtreF* | This study |
| BW3-PLD | [BW3] pBADKP-pld2 | This study |
| BWK | [BW3] *Δkch* |  |
| BWK-PLD | [BWK] pBADKP-pld2 | This study |
| *E. coli* DH5α | *F^-^ endA1 glnV44 thi-1 recA1 relA1 gyrA96 deoR nupG purB20 φ80dlacZΔM15 Δ(lacZYA-argF)U169 hsdR17(r_K_^-^ m_K_^+^)λ^-^* | Transgene |
| Plasmids |  |  |
| Pcas | *repA101(Ts) kan P_cas_-cas9 P_araB_-Red lacI^q^ P_trc_-sgRNA-pMB1* | Lu lab |
| pTargetF | *pMB1 aadA sgRNA* | Lu lab |
| pTtargetF-*kch* | *pMB1 aadA sgRNA-kch* | This study |
| pBADKP-pld2 | Kan^R^，pBR322 origin，P_BAD_. Gene III secretion signal sequence，*par* region，codon-optimized  *S. antibioticus pld* | Lu lab |

Fig. S1 Thin-layer chromatography results of phospholipid standard samples

Fig. S2 PLD expression results
